# Structural Application of Lightweight Panels Made of Waste Cardboard and Beech Veneer

**DOI:** 10.3390/ma14175064

**Published:** 2021-09-04

**Authors:** Vassil Jivkov, Ralitsa Simeonova, Petar Antov, Assia Marinova, Boryana Petrova, Lubos Kristak

**Affiliations:** 1 Department of Interior and Furniture Design, Faculty of Forest Industry, University of Forestry, 1797 Sofia, Bulgaria; r_simeonova@ltu.bg (R.S.); a_marinova@ltu.bg (A.M.); b.petrova@ltu.bg (B.P.); 2 Department of Interior and Architectural Design, Faculty of Architecture, University of Architecture, Civil Engineering and Geodesy, 1046 Sofia, Bulgaria; 3 Department of Mechanical Wood Technology, Faculty of Forest Industry, University of Forestry, 1797 Sofia, Bulgaria; p.antov@ltu.bg; 4 Faculty of Wood Sciences and Technology, Technical University in Zvolen, T. G. Masaryka 24, 960 01 Zvolen, Slovakia

**Keywords:** lightweight panels, waste cardboard, corner joints, bending strength, stiffness

## Abstract

In recent years, the furniture design trends include ensuring ergonomic standards, development of new environmentally friendly materials, optimised use of natural resources, and sustainably increased conversion of waste into value-added products. The circular economy principles require the reuse, recycling or upcycling of materials. The potential of reusing waste corrugated cardboard to produce new lightweight boards suitable for furniture and interior applications was investigated in this work. Two types of multi-layered panels were manufactured in the laboratory from corrugated cardboard and beech veneer, bonded with urea-formaldehyde (UF) resin. Seven types of end corner joints of the created lightweight furniture panels and three conventional honeycomb panels were tested. Bending moments and stiffness coefficients in the compression test were evaluated. The bending strength values of the joints made of waste cardboard and beech veneer exhibited the required strength for application in furniture constructions or as interior elements. The joints made of multi-layer panels with a thickness of 51 mm, joined by dowels, demonstrated the highest bending strength and stiffness values (33.22 N∙m). The joints made of 21 mm thick multi-layer panels and connected with Confirmat had satisfactory bending strength values (10.53 N∙m) and Minifix had the lowest strength values (6.15 N∙m). The highest stiffness values (327 N∙m/rad) were determined for the 50 mm thick cardboard honeycomb panels connected by plastic corner connector and special screw Varianta, and the lowest values for the joints made of 21 mm thick multi-layer panels connected by Confirmat (40 N∙m/rad) and Minifix (43 N∙m/rad), respectively. The application of waste corrugated cardboard as a structural material for furniture and interiors can be improved by further investigations.

## 1. Introduction

Limited wood resources worldwide require efficient utilisation of waste and by-products, cascading use of the available lignocellulosic raw materials, and search for alternative production processes and materials [1,2,3,4,5,6,7]. The COVID-19 crisis has also led to changes in the market and a shortage of resources, including solid wood and furniture panels. The reuse of materials provides new opportunities and represents a sustainable way to address this shortage. The use of corrugated cardboard, a mainstream packaging material, is an option to minimise the problem.

One of the pioneers in the use of non-traditional solutions to save raw materials and lighten the construction of furniture is IKEA. The furniture giant applied paper as a honeycomb core in the 1980s when they started producing the Lack series [8], which was significantly further developed in the last 10–15 years. The honeycomb core is usually prefabricated from craft paper and delivered at an ordered thickness as compressed harmonica-like layers [9]. The technology for producing such panels has been significantly developed over the last 10 years [10]. Panels weighing less than 500 kg/m^3^ are defined as light panels, below 350 kg/m^3^ are very light, and below 200 kg/m^3^ are ultra-light ones [11]. Some of the first comprehensive studies on the application, manufacturing, and properties of paper honeycomb panels were done by Hänel and Weinert [12] and Poppensieker and Thömen [13]. Many studies were made to evaluate the mechanical properties of honeycomb panels [14,15,16,17,18,19,20,21]. Other studies investigated the possibilities for optimisation of the structure of paper honeycomb panels. Smardzewski and Prekrat [22] concluded that the stiffness and strength of cell panels were affected significantly by the weight of paper used to manufacture their cores and the shape and dimensions of cells. Other results showed that sandwich panels with a wavy core can sustain higher loads than honeycombs [23]. Słonina et al. [24] reported that impregnating solutions can be applied to improve the paper core’s stiffness. The possibility of manufacturing lightweight flat pressed wood plastic composites were investigated by Lyutyy et al. [25].

Gößwald et al. [26] investigated the potential of using planer shavings with a length over 4 mm for manufacturing low-density one-layer particleboard with a thickness of 10 mm as an option to reduce the raw material demand for wood-based panels. A team of scientists [27] investigated the effect of relative humidity on the strength properties of lightweight panels and found out that after exposure to 95% relative humidity, facings and sandwiches lost up to half their original strength properties. By establishing a 3D moisture-displacement finite element model, the influence of constant and cycle humidity and varied temperature on the flexural creep of the sandwich panel containing Kraft honeycomb core and wood composite skins was studied by Chen et al. [28]. The influence of core shape was studied by several authors [23,29,30,31]. In the last few years, the strength and stiffness of furniture panels with auxetic cores were investigated. The auxetic cores of the sandwich cellular wood panels exhibited strong orthotropic properties [32]. During bending, wood-based honeycomb panels with auxetic cores absorb energy more effectively than the same panels subjected to axial compression [20]. The relative density of cells significantly affects their mechanical strength [33]. Using an auxetic core and facings of plywood and cardboard significantly reduces the amount of dissipated energy [21].

The problematic parts of applying paper honeycomb panels as structural elements are the joints [11]. For this reason, there is a growing industrial and academic interest on the investigation and optimization of these types of joints [16,34,35,36,37,38]. Some of the authors studied the bending strength of the joints [36,38,39], but tensile and shear tests were applied, as well [15,34,35,38,39]. Lightweight honeycomb panels with different thicknesses have been tested. Several authors studied panels with a relatively small thickness, ranged between 15 and 19 mm [21,22,34,35,38,39,40,41]. Other studies were focused on the evaluation of panels with a thickness of 38 mm [15,34,36]. Only one research was found with a commercially produced honeycomb panel with 50 mm thickness [37]. Both glued [21,34] and non-glued joints [36,37,38] appeared in the studies. Screw withdrawal resistance in honeycomb panels was investigated by some researchers [15,37,40,41].

Lightweight paper panels and paper tubes were analysed concerning their suitability as furniture materials by Petutschnigg and Ebner [16]. The authors concluded that the bending strength of the lightweight paper panel is too low for use in furniture applications and development of materials and joints with enhanced strength properties is needed.

Corrugated cardboard has a relatively low weight, is easily available, recyclable, and waste cardboard can be reused. With the development of online commerce, the use of cardboard packaging, including corrugated cardboard, has been significantly increased. In 2018, the production of paper and paperboard packaging was estimated to 256,138 thousand metric tons worldwide [42]. Around 80% of all goods sold in Europe and the US are in cartons [43]. At the same time, only 65.7% of waste cardboard in the European Union is recycled [44]. This enormous amount of waste cardboard is a challenge to absorb and obtain new products based on recycling or reuse.

In the literature, there are publications about using waste cardboard as a material for producing new panels. In part of the research, the cardboard is processed and incorporated into new composites [45,46]. Several investigations were carried out to evaluate the mechanical properties of cardboard made from recycled beverage cartons [47,48,49,50,51]. Other researchers used the waste cardboard in the form in which it was obtained [52]. There are many examples of furniture made of cardboard, such as shelves, chairs, tables, desks, even sofas [53]. Some companies specialised in the production of cardboard furniture [54,55]. A Japanese bedding company has created a cardboard bed for athletes at the Tokyo 2020 Olympics. The bed frames were made from recycled cardboard [56]. There is also furniture made of second-hand cardboard, but these are usually of relatively simple structure. Even building construction components were objects of experimentation using sheets of waste cardboard collected from the waste stream [57]. Corrugated cardboard has a good strength-to-weight ratio, excellent burst strength, and resistance to crushing, thus being an ideal material for furniture manufacture [58]. Furthermore, adhesives applicable in the wood-processing and furniture industry can be used to bond such panels.

There is limited information about the strength characteristics of the corner joints made of corrugated cardboard.

The aim of the study was to investigate the possibilities of joining lightweight panels made from waste corrugated board and beech veneer, bonded with urea-formaldehyde (UF) resin and evaluate their application in furniture and interior constructions. For strength comparison, joints obtained from conventional lightweight cardboard honeycomb panels with a thickness of 50 mm were also tested.

## 2. Materials and Methods

### 2.1. Materials

In order to establish the possibility of using waste cardboard as a structural material for furniture and interior applications, experimental multi-layer panels were produced in the laboratory using a three-ply corrugated waste cardboard and rotary cut beech (*Fagus sylvatica* L.) veneer sheets with a thickness of 1.2 mm and a moisture content of approximately 8%, provided by the factory Welde Bulgaria AD (Troyan, Bulgaria). The waste cardboard sheets with a length of 1150 mm and width of 780 mm were taken from packages of goods, shipping in pallets, where this cardboard is used as a divider between individual goods. The cardboard sheets had the following dimensions: Thickness of 3.94 mm, top layer of 0.15 mm, and corrugated paper core layer of 0.10 mm. Density of the cardboard was 114 kg/m^3^. Commercially available UF resin with dry solids content of 64%, density of 1.29–1.31 g.cm^−3^ at 20 °C, pH value of 8.5, and a molar ratio of 1.16, provided by the company Kastamonu Bulgaria AD (Gorno Sahrane, Bulgaria), was used for hot pressing. The experimental panels were fabricated under laboratory conditions on a single opening hydraulic press (PMC ST 100, Italy). The press temperature used was t = 100 °C. The specific bonding pressure was 0.2 N/mm^2^ for 21 mm thickness and 0.13 N/mm^2^ for 51 mm thickness of the panels, respectively. 

Commercial, commonly produced lightweight panels with a thickness of 50 mm were also included in the study to compare the obtained experimental data with industrially produced lightweight panels. The top layers were 8 mm laminated particleboards and a hexagon honeycomb for the core layer. The commercial panels were supplied by Egger (Fritz Egger GmbH & Co. OG, Weiberndorf 20, 6380 St. Johann in Tirol, Austria).

**Figure 1:**
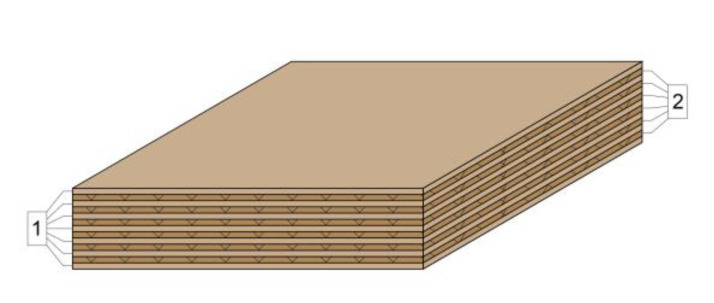
Multi-layer panel with a nominal thickness of 21 mm made of 7 layers of veneer and 6 layers of corrugated cardboard: 1. Rotary cut beech veneer, δ = 1.2 mm; 2. three-layer corrugated cardboard δ_н_ = 6 mm.

**Figure 2:**
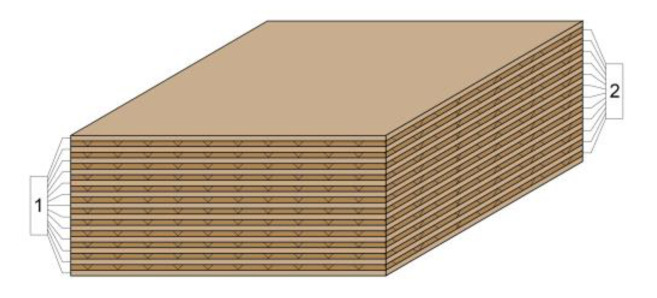
Multi-layer panel with a nominal thickness of 51 mm made of 13 layers of veneer and 12 layers of corrugated cardboard: 1. Rotary cut beech veneer, δ = 1.2 mm; 2. three-layer corrugated cardboard δ_н_ = 6 mm.

**Figure 3:**
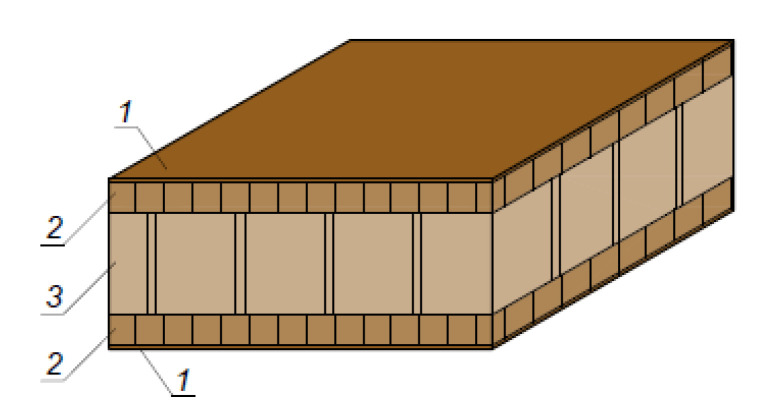
Cardboard honeycomb panel with a thickness of 50 mm, manufactured by Egger, Austria, δ = 50 mm: 1. Decorative foil; 2. PB, δ = 8 mm; 3. honeycomb cardboard core.

The laboratory-fabricated multi-layer panels were designed with thicknesses similar to the thicknesses of the commercially available panels, i.e., 50 and 20 mm. This was achieved by adjusting the bonding pressure. The structure of the lightweight panels used in the study was as follows:

1. Multi-layer panel with a thickness of 21 mm made of 7 layers of veneer and 6 layers of corrugated cardboard (Figure 1).
2. Multi-layer panel with a thickness of 51 mm made of 13 layers of veneer and 12 layers of corrugated cardboard (Figure 2).
3. Cardboard honeycomb panel with a thickness of 50 mm, manufactured by Egger, Austria (Figure 3).

After hot pressing, the laboratory-produced panels were conditioned for 7 days at 20 ± 2 °C and 65% relative humidity. 

The density of all fabricated panels was below 500 kg/m^3^ (Table 1). Details about the manufacturing process and the physical and mechanical properties of the developed lightweight panels are given in a previous research [18]. 

### 2.2. Type of Corner Joints Made of Lightweight Panels

**Figure 4:**
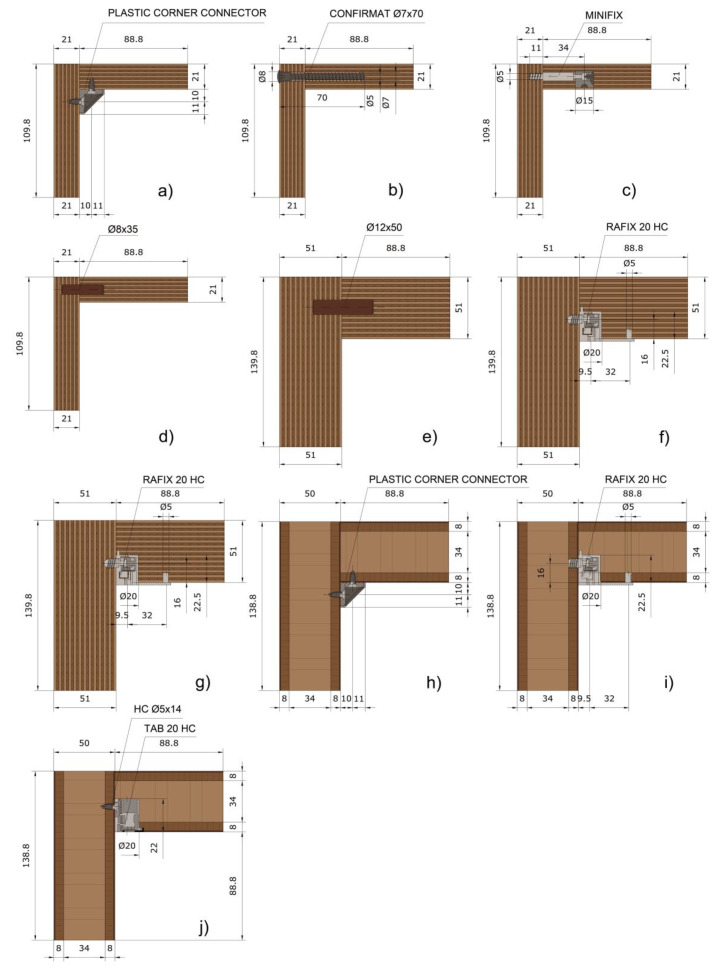
Corner joints made of lightweight panels: (**a**) Multi-layer panel (21 mm) made of veneer and corrugated cardboard, connected by a plastic corner connector and special screw Varianta; (**b**) multi-layer panel (21 mm) made of veneer and corrugated cardboard, connected by Confirmat ø7 × 70 mm; (**c**) multi-layer panel (21 mm) made of veneer and corrugated cardboard, connected by an eccentric connector Minifix; (**d**) multi-layer panel (21 mm) made of veneer and corrugated cardboard, connected by a dowel ø8 × 35 mm; (**e**) multi-layer panel (51 mm) made of veneer and corrugated cardboard, connected by a dowel ø12 × 50 mm; (**f**) multi-layer panel (51 mm) made of veneer and corrugated cardboard, connected by Rafix 20 HC, inserted longitudinally on the cardboard direction; (**g**) multi-layer panel (51 mm) made of veneer and corrugated cardboard, connected by Rafix 20 HC, inserted across on the cardboard direction; (**h**) cardboard honeycomb panel (50 mm) connected by plastic corner connector and special screw Varianta; (**i**) cardboard honeycomb panel (50 mm) connected by Rafix 20 HC; (**j**) cardboard honeycomb panel (50 mm) connected by TAB 20 HC.

For the purpose of the study, 10 series with a total number of 129 test samples were made. Some well-known connecting solutions were used for the joints, such as a dowel joint, one-element connector Confirmat, an eccentric connector Minifix with a bolt for ø5 mm hole and a plastic corner joint, as well as special connectors and screws designed for lightweight honeycomb panels. Joint types, detailed dimensions, and types of connecting elements are shown in Figure 4. Each joint had only one connecting element.

### 2.3. Test Methods

**Figure 5:**
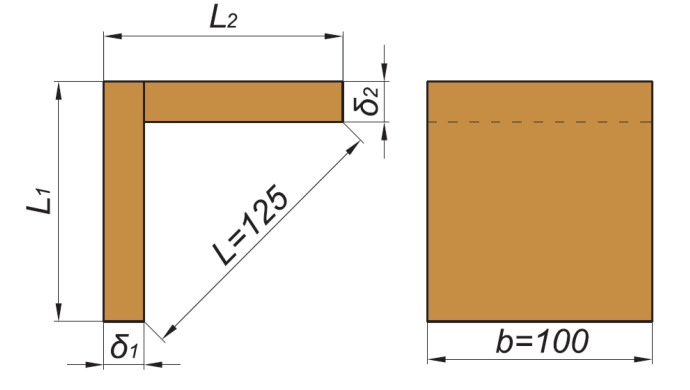
Type and dimensions of the tested samples. Reprinted with permission from Ref. [11]. 2021 BISMAR.

The type and shape of test samples, made in accordance with the test method, described by Kyuchukov and Jivkov [11], are shown in Figure 5. The dimensions δ_1_ and δ_2_ are equal and correspond to the thickness of the panels. The dimensions L_1_ and L_2_ are also equal and depend on the thickness of the panels.

**Figure 6:**
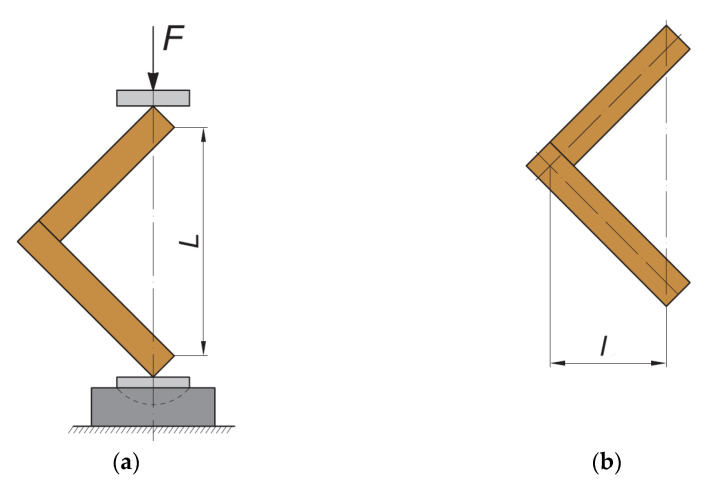
Test procedure. Reprinted with permission from Ref. [11]. 2021 BISMAR. (**a**) Type of loading of the tested samples; (**b**) determination of the bending arm.

In furniture constructions, the joints are most often loaded in bending with arm compression and arm opening. It has been found from many research studies that the strength and the deformation resistance of the joints are lower under the arm compression loading compared to that under loading with arm opening [11]. Therefore, for the purpose of the present study, it was accepted to investigate end corner joints of lightweight structural elements under bending loading with arm compression. The principal test scheme of the test specimens is given in Figure 6.

The criterion for determining the strength of the tested joints is the maximum bending moment *M_max_* calculated according to the formula:

*M_max_* = *F* ∙ *l*, (1)

where *F* is the maximum force under arm compression bending, *N*, and *l* is arm, m.

The criterion for determining the deformation characteristic of the corner joints is the stiffness coefficient *с* [11,59].

**Figure 7:**
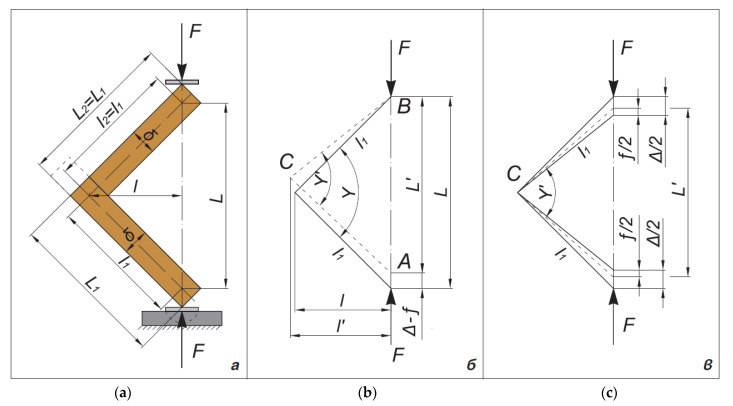
Test scheme and deformation under arm compression bending load of the test samples of end corner joints made of lightweight panels. Reprinted with permission from Ref. [11]. 2021 BISMAR. (**a**) Type of loading and dimensions of the tested samples; (**b**,**c**) scheme of loading and determination of the deformation of the tested samples.

The deformation of the joints under the compression bending test gives as a result changes in both the right angle between the joint arms and the bending arms *l* of the forces (Figure 7). 

The linear displacement *f_i_* of the application points of the forces *F_i_* is recorded for each test sample at each loading level. It represents a sum of displacement resulting from turning the joint arms and additional displacement Δ*_i_* resulting from bending of the arms.

The displacement Δ*_i_* is calculated by the formula:

(2) $$\Delta_{i}=\frac{F_{i}a^{3}}{3EI}$$

where

*F_i_* is the magnitude of the load forces with arm compression, N;

*a* is the axial length of the joint arms, m;

*E* is the modulus of elasticity, N/mm^2^;

*I* is the axial moment of inertia of the cross-section of the joint arms, m^4^,

which is calculated by the formula:

(3) $$I=\frac{\delta b^{3}}{12},$$

where

*b* is the width of the arms, m;

*δ* is the thickness of the arms, m.

The distance between the force application points at each level of loading is determined by the formula:

(4) $$L_{1}=L-f_{i}+\Delta_{i},$$

The angle $\gamma_{i}$ [rad] changed under loading between the joint arms is calculated by the formula:

(5) $$\gamma_{i}=2\arcsin\frac{L_{i}}{2a}=2\arcsin\frac{L-f_{i}+\Delta_{i}}{2a},$$

The changed bending arm *l_i_* is determined by the formula:

(6) $$l_{i}=a\cos\frac{\gamma_{i}}{2},$$

The result from the deformation under the compression bending test is the semi-rigid rotation of the joint arms in [rad]:

(7) $$\alpha_{i}=\frac{\pi }{2}-\gamma_{i},$$

For 10 and 40% of the load force *F_i_*, the bending moment in [N∙m] is calculated according to the formula:

(8) $$M_{i}=F_{i}l_{i},$$

The stiffness coefficient under the compression bending test *c_i_* [N∙m/rad] is calculated by the formula:

(9) $$c_{i}=\frac{\Delta M_{i}}{\Delta \alpha_{i}},$$

In (8), the following designations are used:

$$\Delta M_{i}=M_{i}-M_{0}$$

$$\Delta \alpha_{i}=\alpha_{i}-\alpha_{0}$$

where *M_i_* and *α_i_* are determined according to (8) and (7) for the value of force *F_i_* equal to 40% of *F_max_*, and *M*_0_ and *α*_0_ according to (8) and (7) or the value of force *F*_0_, equal to 10% of *F_max_*.

The stiffness coefficient *c* as a deformation characteristic of the corner joint under the compression bending test is defined as the arithmetic means of the result of (9) numbers for each test sample when loaded in the section, which corresponds to the linear section on the curve of the correlation between the bending moment and the corner deformation of the joint.

The test was carried out in the scientific and research laboratory at the Institute of Mechanics and Biomechanics, BAS, Sofia, on a TIRA test 2000 universal type testing machine (TIRA GmbH, Schalkau, Germany). A sensor with a range of 1 kN was used to measure the force. The accuracy was 1%, for force over 10 N. The exact speed of the machine’s moving traverse was measured with the aid of the built-in incremental displacement measurement system and an electronic stopwatch. Using a Protek D470 handheld digital multi-meter (Protek Instrument Co., Ltd, Gwangmyeong Gwangmyeong, Korea) and a computer, the force-time relationship was recorded. The traverse displacement was calculated according to time and speed. In determining the deflection of the test sample, the deflection of the loaded parts of the machine was taken into account. The resulting force-displacement diagrams were centred. The maximum force, deflection at the maximum force, force and deflection at a point taken as the failure point, *F_f_* = 0.8*F_max_*, were determined. Tests were carried out at the temperature of 20 ± 1 °C and a relative humidity of 55 ± 5%.

Descriptive statistical analysis of the results was done with XLSTAT, version 2020.2.3, Addinsoft, New York, NY, USA (2021). One-way ANOVA was performed on the results for the bending strength and stiffness coefficient of L-type corner joints to analyse variance at a 95% confidence interval (*p* < 0.05). The statistical differences between mean values were evaluated using Tukey’s honestly significant difference (HSD) post hoc test.

## 3. Results

### 3.1. Bending Moments of Corner Joints Made of Lightweight Panels

**Figure 8:**
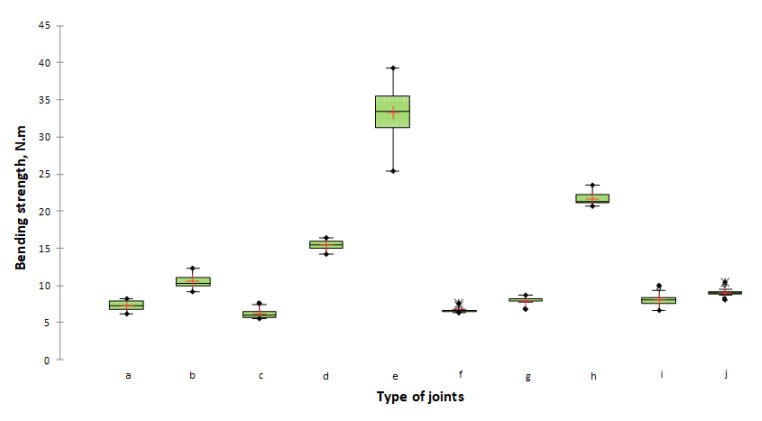
Bending strength under the compression test of L-type corner joints made of lightweight panels. The letter index of the type of joints is according to Figure 4.

The results obtained from the bending strength test were processed statistically and are presented in Table 2 and Figure 8.

In analysing the results for the bending strength test of corner L-type joints of lightweight panels, it can be seen that they vary over a fairly wide range, from 33.33 to 6.15 N∙m. From the statistical analysis of the one-way ANOVA test and the pairwise comparison performed with the Tukey HSD, a significant difference of α = 0.05 at a confidence level of 95%, was found in seven groups between the obtained bending strength of the L-type end corner joints constructed from lightweight panels. The groups are given in Table 3. The highest strength value of 33.22 N∙m was determined for the joints made of multi-layer veneer panels and three-layer cardboard with a nominal thickness of 51 mm and joined by a dowel ø12 × 50 mm. This result can be attributed to the application of adhesive also on the edge of the glued workpiece and the large thickness of the structural elements. The cardboard honeycomb panel (50 mm) connected by a plastic corner connector and special screw Varianta exhibited a bending strength of 21.66 N∙m. The multi-layer panel (21 mm) made of veneer and corrugated cardboard, connected by a dowel ø8 × 35 mm, also showed excellent strength characteristics (15.41 N∙m). 

The remaining joints demonstrated satisfactory strength characteristics with bending strength values ranging from 6.15 to 10.53 N.m. In this group, the highest strength had series *b*—multi-layer panel (21 mm) made of veneer and corrugated cardboard, connected by Confirmat ø7 × 70 mm (10.53 N∙m), followed by series *j*—cardboard honeycomb panel (50 mm) connected by TAB 20 HC (9.06 N∙m), and series *i*—cardboard honeycomb panel (50 mm) connected by Rafix 20 HC (8.10 N∙m). Finally, the lowest bending strength of 6.15 N∙m was recorded in series *c*—multi-layer panel (21 mm) made of veneer and corrugated cardboard, connected by an eccentric connector Minifix.

### 3.2. Stiffness Characteristics of Corner Joints Made of Lightweight Panels

**Figure 9:**
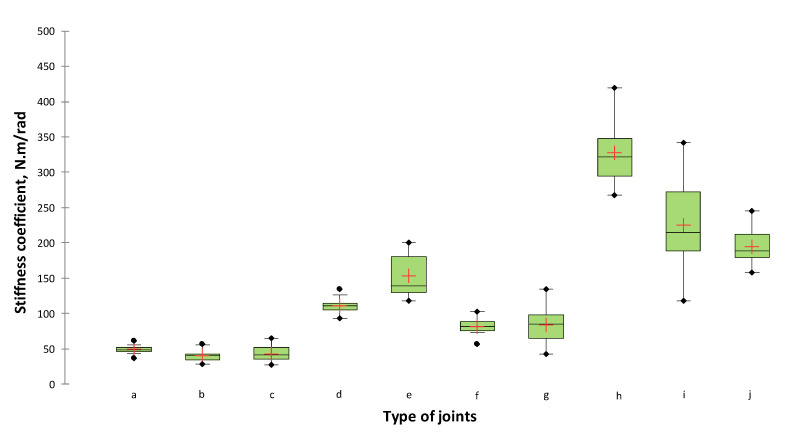
Stiffness coefficients under the compression test of L-type corner joints made of lightweight panels. The letter index of the type of joints is according to Figure 4.

When analysing the results for the stiffness of the corner L-type joints of lightweight panels, it can be seen that the picture was slightly different. The stiffness values varied from 327 to 40 N∙m/rad (Table 4). From the statistical analysis of the one-way ANOVA test and the pairwise comparison performed with the Tukey HSD (Table 5), a significant difference of α = 0.05 at a confidence level of 95% was found in six groups between the obtained stiffness coefficients L-type end corner joints constructed from lightweight panels. The greatest stiffness was exhibited by joints made of conventional honeycomb lightweight 50 mm thick panels. This can be explained with an outer layer that is 8 mm thick, which gives more rigidity to the panel and joints. The highest stiffness value of 327 N∙m/rad had the joints made of honeycomb panel—series *h*, connected by a plastic corner connector and special screw Varianta, followed by a cardboard honeycomb panel (50 mm) connected by Rafix 20 HC (225 N∙m/rad), and a cardboard honeycomb panel (50 mm) connected by TAB 20 HC (195 N∙m/rad). A multi-layer panel (51 mm) made of veneer and corrugated cardboard, connected by a dowel ø12 × 50 mm was the joint with the highest stiffness of the corrugated board joints (154 N∙m/rad), followed by a multi-layer panel (21 mm) made of veneer and corrugated cardboard, connected by Confirmat ø7 × 70 mm (111 N∙m/rad). A very low stiffness was shown by the joints made of a multi-layer panel made of veneer and corrugated cardboard with a thickness of 21 mm. Joints from these panels connected by a plastic corner connector and special screw Varianta had a stiffness coefficient of 49 N∙m/rad, followed by an eccentric connector Minifix (43 N∙m/rad) and Confirmat ø7 × 70 mm (40 N∙m/rad). A graphical representation of the results obtained is shown in Figure 9. 

## 4. Discussion

### 4.1. Bending Strength of Corner Joints Made of Lightweight Panels

From the results obtained for the bending strength of the corner joints, made of lightweight panels comprising waste cardboard and beech veneer, bonded with UF resin, it can be concluded that they possess the required strength for application in furniture constructions or as interior elements. The joints in both variants of multi-layer corrugated cardboard, with a thickness of 21 and 51 mm, exhibited good bending strength characteristics when connected by gluing with dowels. The results were within the range of bending strength values achieved by [36], where, however, the authors used two connecting elements in each joint. Joints of cardboard honeycomb panel (50 mm) connected by a plastic corner connector and special screw Varianta showed excellent bending strength. More than twice is the strength exhibited by the joints with TAB 20HC in the present study compared to the study conducted by Joscak et al. [60]. To note, multi-layer panels with a thickness of 21 mm made of veneer and corrugated cardboard, connected by an eccentric connector Minifix, showed lesser strength compared to the results of other studies [38,61]. However, it was in the range of similar joints made of laminated particleboards and medium-density fiberboard (MDF) with a thickness of 18 mm [11,59]. There was no significant difference in the joint’s strength of multi-layer panel (51 mm) made of veneer and corrugated cardboard, connected by Rafix 20 HC, inserted across and longitudinally on the cardboard direction. Cardboard honeycomb panel (50 mm) and multi-layer panel (51 mm) made of veneer and corrugated cardboard connected by Rafix 20 HC shows a similar joint strength. Although the bending strength of the joints of 21 mm thick multi-layer panels made of waste cardboard and beech veneer connected by Confirmat did not reach the values obtained with particleboards, plywood, and MDF [62,63,64,65,66,67], still their strength properties were sufficient to be used as an option for joining.

### 4.2. Stiffness Characteristics of Corner Joints Made of Lightweight Panels

When analysing the results for the stiffness of the joints, it can be seen that the picture is different. The highest stiffness values were obtained for the joints made of 50 mm thick cardboard honeycomb panel coupled by a plastic corner connector with a special screw Varianta, Rafix 20 HC, and TAB 20 HC, and might be explained by the excellent stiffness of the outer layers of laminated chipboard and the good construction of the special connectors and screws developed for honeycomb panels. In the present study, joints connected with TAB 20 HC showed significantly higher stiffness compared to the results obtained by [37].

In contrast to the bending strength of the joints, the stiffness of the joints of cardboard honeycomb panel (50 mm) is more than two times higher compared to the multi-layer panel (51 mm) made of veneer and corrugated cardboard when connected by Rafix 20 HC.

Both multi-layer panels made of veneer and corrugated cardboard with a thickness of 51 and 21 mm, joined with a dowel ø12 × 50 mm and dowel ø8 × 35 mm, respectively, had sufficient rigidity to be used in furniture construction due to the application of an adhesive. Furthermore, the stiffness coefficients were in the range of other commonly used joints [68,69,70,71,72,73].

Joints exhibited relatively low stiffness with Confirmat and fasteners where screws were involved. The low stiffness of the 21 mm thick multi-layer veneer and corrugated cardboard joints resulted from the material’s lack of a rigid and dense structure, which prevented a high screw-holding capacity.

## 5. Conclusions

The COVID-19 pandemic posed new challenges to the normal business practice and competitiveness of industrial enterprises, connected with significant changes in the market and shortage of resources, including solid wood and furniture panels [74,75,76,77]. This study investigated the potential of using waste corrugated cardboard and beech veneer for manufacturing lightweight panels for furniture and interior structural elements application. For this purpose, various joints, fixed with adhesive and demountable joints were tested. As a result, the following conclusions were drawn:

1. The waste corrugated cardboard in combination with beech veneer is suitable for furniture constructions and interior elements.
2. The use of adhesive significantly increased the bending strength and stiffness of the joints of the veneer and corrugated cardboard panels in both studied panel thicknesses, i.e., 21 and 51 mm.
3. In terms of the stiffness coefficients, the best behaviour was exhibited by the joints, made of cardboard honeycomb panels with a thickness of 50 mm.
4. Except for the cardboard honeycomb panels, all of the tested panels where the demountable joints were used, showed a significantly lower strength compared to bonding.
5. There was no significant difference in the joint strength of the multi-layer 51 mm thick panel made of veneer and corrugated cardboard, connected by Rafix 20 HC, inserted across and longitudinally on the cardboard direction.
6. The cardboard honeycomb panel (50 mm) and multi-layer panel (51 mm) made of veneer and corrugated cardboard connected by Rafix 20 HC show a similar joint strength.
7. Although lightweight corrugated cardboards did not have sufficient density, the conventional joints such as Minifix, Confrimat, and plastic corner joints demonstrated comparable strength characteristics and stiffness to particleboard joints.

Future research should be focused on the development of the multi-layer waste corrugated cardboard and veneer lightweight panels as a prospective and sustainable material for furniture and other applications, in order to improve the strength and other features of the joints.

## Figures and Tables

**Table 1:** Thickness and density of the lightweight panels.

Type of Panel	Thickness, mm	Density kg/m^3^
1. Multi-layer panel, veneer (7 layers) and corrugated cardboard (6 layers)	21	466
2. Multi-layer panel, veneer (13 layers) and corrugated cardboard (12 layers)	51	349
3. Cardboard honeycomb panel	50	257

**Table 2:** Bending moments of corner joints made of lightweight panels.

Type of Joints ^1^	No. of Test Samples	Mean, N∙m	Min., N∙m	Max., N∙m	Median, N∙m	St. Dev., N∙m	Var. Coefficient
a	15	7.28	6.10	8.22	7.25	0.64	0.09
b	15	10.53	9.21	12.25	10.29	0.82	0.08
c	15	6.15	5.49	7.52	5.97	0.61	0.10
d	15	15.41	14.20	16.41	15.42	0.62	0.04
e	8	33.22	25.39	39.33	33.46	4.01	0.12
f	10	6.70	6.28	7.58	6.56	0.39	0.06
g	7	7.90	6.70	8.59	7.89	0.56	0.07
h	14	21.66	20.66	23.52	21.33	0.85	0.04
i	15	8.10	6.68	9.91	8.09	0.87	0.11
j	14	9.06	8.05	10.34	8.99	0.59	0.07

^1^ The letter index of the type of joints is according to Figure 4.

**Table 3:** Tukey HSD analysis of the differences between the groups with a confidence interval of 95% of bending capacity of joints of two materials of all pairwise comparisons.

Index ^1^	Type of Joint	Bending Strength N∙m	Lower Bound (95%)	Upper Bound (95%)	GH ^2^
e	Multi-layer panel (51 mm) made of veneer and corrugated cardboard, connected by a dowel ø12 × 50 mm	33.22	32.34	34.10	A
h	Cardboard honeycomb panel (50 mm) connected by plastic corner connector and special screw Varianta	21.66	21.13	22.18	B
d	Multi-layer panel (21 mm) made of veneer and corrugated cardboard, connected by a dowel ø8 × 35 mm	15.41	14.76	16.05	C
b	Multi-layer panel (21 mm) made of veneer and corrugated cardboard, connected by Confirmat ø7 × 70 mm	10.53	10.03	11.04	D
j	Cardboard honeycomb panel (50 mm) connected by TAB 20 HC	9.06	8.42	9.70	DE
i	Cardboard honeycomb panel (50 mm) connected by Rafix 20 HC	8.10	7.46	8.74	EF
g	Multi-layer panel (51 mm) made of veneer and corrugated cardboard, connected by Rafix 20 HC, inserted across on the cardboard direction	7.90	6.96	8.41	EFG
a	Multi-layer panel (21 mm) made of veneer and corrugated cardboard, connected by a plastic corner connector and special screw Varianta	7.28	6.33	7.91	FG
f	Multi-layer panel (51 mm) made of veneer and corrugated cardboard, connected by Rafix 20 HC, inserted longitudinally on the cardboard direction	6.70	5.90	7.56	FG
c	Multi-layer panel (21 mm) made of veneer and corrugated cardboard, connected by an eccentric connector Minifix	6.15	5.64	6.65	G

^1^ The letter index of the type of joints is according to Figure 4. ^2^ Groups of homogeneities (*α* = 0.05).

**Table 4:** Stiffness coefficients of corner joints made of lightweight panels.

Type of Joints ^1^	No. of Test Samples	Mean, N∙m/Rad	Min., N∙m/Rad	Max., N∙M/Rad	Median, N∙M/Rad	St. Dev., N∙m/Rad	Var. Coefficient
a	15	49.19	36.68	61.40	48.77	5.43	0.11
b	15	40.28	27.81	57.14	40.09	7.83	0.19
c	15	42.72	27.46	65.32	41.23	10.56	0.25
d	15	111.02	92.99	134.05	110.47	9.92	0.09
e	8	153.61	117.33	200.91	139.03	31.41	0.20
f	10	81.27	56.86	102.07	81.77	11.62	0.14
g	7	83.76	42.50	134.66	84.69	28.59	0.34
h	14	327.34	267.24	419.56	321.33	39.26	0.12
i	15	224.61	117.49	342.42	215.01	58.87	0.26
j	15	194.71	157.93	245.26	188.06	24.97	0.13

^1^ The letter index of the type of joints is according to Figure 4.

**Table 5:** Tukey HSD analysis of the differences between the groups with a confidence interval of 95% of stiffness coefficients of joints of two materials of all pairwise comparisons.

Index ^1^	Type of Joint	Stiffness Coeff. c, N∙m/rad	Lower Bound (95%) N∙m/rad	Upper Bound (95%) N∙m/rad	GH ^2^
h	Cardboard honeycomb panel (50 mm) connected by a plastic corner connector and special screw Varianta	327	311.79	342.89	A
i	Cardboard honeycomb panel (50 mm) connected by Rafix 20 HC	225	209.59	239.64	B
j	Cardboard honeycomb panel (50 mm) connected by TAB 20 HC	195	177.58	208.68	BC
e	Multi-layer panel (51 mm) made of veneer and corrugated cardboard, connected by a dowel ø12 × 50 mm	154	133.04	174.19	C
d	Multi-layer panel (21 mm) made of veneer and corrugated cardboard, connected by a dowel ø8 × 35 mm	111	96.00	126.05	D
g	Multi-layer panel (51 mm) made of veneer and corrugated cardboard, connected by Rafix 20 HC, inserted across on the cardboard direction	84	61.77	105.76	DE
f	Multi-layer panel (51 mm) made of veneer and corrugated cardboard, connected by Rafix 20 HC, inserted longitudinally on the cardboard direction	81	62.87	99.67	DE
a	Multi-layer panel (21 mm) made of veneer and corrugated cardboard, connected by a plastic corner connector and special screw Varianta	49	34.17	64.21	EF
c	Multi-layer panel (21 mm) made of veneer and corrugated cardboard, connected by an eccentric connector Minifix	43	27.70	57.74	EF
b	Multi-layer panel (21 mm) made of veneer and corrugated cardboard, connected by Confirmat ø7 × 70 mm	40	25.26	55.31	E

^1^ The letter index of the type of joints is according to Figure 4. ^2^ Groups of homogeneities (*α* = 0.05).

## Data Availability

Not applicable.
